# The Centiloid Scale in Amyloid PET Imaging: Current Role in Alzheimer’s Disease Diagnosis, Treatment Planning, and Monitoring During Anti-Amyloid Therapy: A Clinical Perspective

**DOI:** 10.3390/diagnostics16131989

**Published:** 2026-06-26

**Authors:** Houman Sotoudeh, Mohammadreza Alizadeh

**Affiliations:** 1 Neuroradiology Section, Department of Radiology, UT Southwestern Medical Center, Dallas, TX 75390, USA; 2 Department of Medicine, Division of Neurology, Faculty of Medicine, The University of British Columbia, Vancouver, BC V5Z 1M9, Canada; danial.alizadeh@vch.ca

**Keywords:** Alzheimer’s disease, amyloid PET, Centiloid scale, lecanemab, donanemab, amyloid-related imaging abnormalities (ARIA), disease-modifying therapy, quantitative neuroimaging, anti-amyloid therapy, biomarker

## Abstract

Amyloid positron emission tomography (PET) has become a critical tool in the diagnosis and treatment of Alzheimer’s disease (AD). The Centiloid (CL) scale, a tracer/scanner-independent, standardized quantification unit introduced in 2015, transforms tracer- and scanner-specific standardized uptake value ratios (SUVRs) into a common metric anchored at 0 CL in young cognitively unimpaired individuals and 100 CL in patients with mild-to-moderate AD. This review synthesizes current evidence on the clinical role of the CL scale across three domains: (1) diagnostic classification, with established thresholds of <10 CL for amyloid negativity and >30 CL for high-certainty amyloid positivity; (2) treatment eligibility, where a 2024 Alzheimer’s Association Research Roundtable consensus of global experts recommended a 24–30 CL threshold for initiating lecanemab or donanemab therapy in patients with mild cognitive impairment (MCI) or mild AD dementia; and (3) longitudinal therapy monitoring, in which serial CL measurements provide objective evidence of amyloid clearance. We also review the emerging ‘gray zone’ (10–30 CL) as a distinct clinical entity with elevated progression risk, the critical role of CL quantification in complementing visual reads in borderline cases, technical limitations, and the future integration of CL in clinical practice. This review also critically addresses the ongoing debate on whether amyloid clearance represents a reliable surrogate for clinical benefit, strategies for managing discordant biomarker findings, and the practical feasibility of serial amyloid PET in routine care. With FDA approval of both lecanemab and donanemab, familiarity with the CL scale as a functional treatment biomarker is increasingly relevant for neuroradiologists and nuclear medicine physicians in the modern AD care pathway. As with all imaging modalities, the CL has physiologic and technical limitations. Although the CL scale was designed to reduce heterogeneity across tracers and scanner platforms, the impact of different commercial quantification software packages on CL output remains incompletely characterized. Consistent use of a single software platform for longitudinal monitoring in individual patients is therefore recommended.

## 1. Introduction

57 million people worldwide are currently living with Alzheimer’s disease (AD), establishing it as the predominant cause of dementia. This figure is expected to climb to 152 million by 2050 as populations continue to age [1]. Yet, the assessment of cognitive symptoms and the exclusion of alternative causes have been the main pillars of its diagnosis for the past decades. This approach has been substantially reconfigured by the advent of molecular biomarkers and the biologically grounded reclassification of AD within the amyloid/tau/neurodegeneration (A/T/N) framework. This characterizes the disorder through the presence of pathophysiological hallmarks rather than solely clinical manifestations [2].

One of the most distinctive biomarkers is amyloid positron emission tomography (PET). Its uniqueness comes from the in vivo, non-invasive quantitative visualization of fibrillar amyloid-beta plaques (Aβ), which permits diagnosis years before the emergence of clinical symptoms [3,4]. Three 18F-labeled tracers, namely florbetapir (Amyvid™), florbetaben (NeuraCeq™), and flutemetamol (Vizamyl™), are now approved by the U.S. Food and Drug Administration (FDA) and the European Medicines Agency (EMA). Consequently, they are now incorporated into routine clinical practice for the diagnostic evaluation of patients with suspected AD [5].

This expanded further with FDA approval of lecanemab (Leqembi™) in 2023 and donanemab (Kisunla™) in July 2024 as the first AD-modifying anti-amyloid monoclonal antibodies capable of slowing cognitive decline. These agents’ initiation strategy begins with verification of amyloid pathology, established through PET imaging or cerebrospinal fluid (CSF) biomarker assessment. This requirement has transformed precise, standardized amyloid quantification from a purely scholarly undertaking into a clinical imperative. However, the precise thresholds, clinical applicability, and relationship between amyloid clearance and cognitive outcomes remain subjects of active debate, as addressed in this review [6,7].

In addition, inter-tracer and inter-site variability impede this measurement. Tracer kinetics, the reference region employed, and the processing pipeline used for these tracers result in different standardized uptake value ratios (SUVRs). For instance, we cannot directly equate a particular SUVR derived from a florbetapir scan with one obtained from a flutemetamol scan [8].

This tracer-agnostic standardization approach linearly rescales each tracer’s SUVR into universal CL units anchored to reference populations of young healthy adults and patients with mild-to-moderate AD. Since its first introduction, the CL scale has been referenced in more than 330 publications and is currently integrated into commercially available quantification systems cleared under FDA 510(k) and bearing CE marking [9].

This review presents a clinically focused summary of CL quantification across its diagnostic, therapeutic, and monitoring roles.

### Review Approach

We conducted a targeted narrative clinical review. We searched PubMed, including MEDLINE, and Embase for English-language publications from 2014 to 2026 using combinations of the terms “Centiloid”, “amyloid PET”, “Alzheimer’s disease”, “amyloid quantification”, “lecanemab”, “donanemab”, “anti-amyloid therapy”, “treatment eligibility”, “amyloid clearance”, and “biomarker discordance”. Reference lists of relevant reviews, consensus statements, appropriate-use criteria, pivotal clinical trials, and methodological papers were also manually reviewed.

We prioritized original clinical studies, neuropathological validation studies, longitudinal cohort studies, pivotal therapeutic trials, consensus statements, and clinically relevant reviews. Publications were excluded if they did not address amyloid PET, Centiloid quantification, anti-amyloid treatment eligibility, treatment monitoring, or biomarker interpretation. Non-English publications were excluded because formal translation resources were not available.

Given the narrative design, we did not perform duplicate independent screening, formal risk-of-bias assessment, meta-analysis, or complete enumeration of all identified and excluded records. When studies reported conflicting thresholds or discordant biomarker findings, we summarized the evidence narratively and distinguished prospective data, retrospective cohort data, neuropathological validation, trial evidence, and expert consensus.

## 2. Biological Basis of Amyloid PET Imaging

### 2.1. Amyloid-Beta Pathophysiology in Alzheimer’s Disease

The characteristic neuropathological feature of AD is the extracellular accumulation of fibrillar amyloid-beta (Aβ) plaques. These deposits are predominantly composed of 40- and 42-amino acid peptides generated by the sequential proteolysis of amyloid precursor protein (APP) via beta- and gamma-secretases [10]. According to the amyloid cascade hypothesis, deposition of aggregated Aβ triggers a sequence of downstream processes, including tau hyperphosphorylation, formation of neurofibrillary tangles, synaptic impairment, and neuroinflammation. This ultimately leads to neuronal degeneration and consequent cognitive decline [11]. Longitudinal imaging and biomarker studies pinpoint amyloid accumulation as preceding clinical symptom onset by 15–20 years, with the earliest in vivo PET signal observed in the precuneus and posterior cingulate cortex, followed by the prefrontal cortex, and subsequent extension to the lateral temporal, parietal, striatal, and ultimately primary sensorimotor cortices in advanced stages of disease [12,13].

### 2.2. FDA-Approved Amyloid PET Tracers

At present, three FDA-approved 18F-labeled radiotracers for clinical amyloid PET imaging are available, all of which bind fibrillar Aβ plaques with high affinity and comparable diagnostic accuracy. However, they diverge in their kinetic properties, preferred acquisition windows, and SUVR reference regions (Table 1).

The findings of the scan can be visually interpreted through gray-white matter contrast. As such, preserved differentiation with tracer retention confined to white matter indicates a negative scan, whereas a positive scan demonstrates loss of this contrast, with cortical ribbon uptake and posterior cingulate involvement in early disease. All three tracers demonstrate strong clinical concordance, with approximately 90% sensitivity and specificity relative to histopathological reference standards [14,15]. Nevertheless, these radiotracers produce systematically divergent SUVR values at an equivalent degree of amyloid burden, thereby preventing direct inter-tracer comparison and necessitating standardization through the Centiloid framework.

## 3. The Centiloid Scale: Technical Foundation

### 3.1. Historical Development

In an attempt in 2015 to overcome cross-tracer and cross-site comparability issues associated with conventional tracer-specific SUVRs, Klunk and colleagues published the seminal paper introducing the Centiloid Project and its methodology, which has since been adopted globally. The project established a dataset of young cognitively normal adults and patients with mild to moderate AD dementia scanned with 11C-Pittsburgh Compound B (PiB). This is the earliest and most comprehensively validated amyloid PET radiotracer, which functions as the reference standard. Each subsequent tracer is calibrated to PiB using conversion equations derived from within-subject, cross-tracer imaging studies [8].

### 3.2. Calculation Methodology

Four sequential steps are used to generate a Centiloid value. First, an amyloid PET scan is acquired using any approved tracer. Second, the tracer-specific SUVR is derived from a cortical composite region of interest and normalized to the appropriate reference region, most commonly the whole cerebellum, or white matter in the case of flutemetamol. Third, a validated tracer-specific linear conversion equation is applied to transform the SUVR into CL units, calibrated against PiB. Finally, a standardized CL value is reported on the 0–100 scale. The mathematical transformation is represented by the equation CL = a × SUVR + b, where a and b denote tracer-specific constants determined empirically through direct comparative validation studies across tracers [16,17,18].

### 3.3. Reference Points and Scale Interpretation

The CL scale is established by two reference benchmarks: 0 CL denotes the average amyloid burden in young, cognitively intact individuals (generally younger than 40 years), whereas 100 CL denotes the average burden identified in patients with mild-to-moderate AD. Although the scale is not restricted to an upper boundary of 100, advanced stages of disease can demonstrate values exceeding this level. In addition, negative values are possible [8,17].

In contrast to SUVR, CL is dimensionless yet biologically interpretable. Each unit represents an estimation of proportional increment in amyloid plaque load relative to the AD reference standard, making the scale more straightforward to interpret in clinical settings. It also has validated thresholds for meaningful change: once between-session measurement variability is considered, a reliable change generally exceeds 10–15 CL, whereas reliable amyloid accumulation is typically defined as greater than 3 CL per year [19].

## 4. Centiloid Thresholds in Alzheimer’s Disease Diagnosis

### 4.1. Established Binary Thresholds

The AMYPAD (Amyloid Imaging to Prevent Alzheimer’s Disease) Consortium has established the most extensively referenced diagnostic paradigm for CL interpretation. This major European research initiative has brought together findings from neuropathological validation studies, visual assessment concordance analyses, and longitudinal cohort data [9]. Their work established two CL cutoffs that demarcate zones of high clinical certainty.

Neuropathological validation studies have established that values below 10 CL are robust markers of the absence of pathologic amyloid-beta deposition. This threshold was validated against Consortium to Establish a Registry for Alzheimer’s Disease (CERAD) neuropathological scoring and Thal amyloid phase staging, with a nearly complete absence of neuritic plaques. Clinically, this range effectively rules out significant amyloid pathology, consistent with levels observed in healthy young controls and in individuals unlikely to derive benefit from anti-amyloid therapy.

Consistently replicated across independent neuropathological cohorts, values exceeding 30 CL have been found highly predictive of underlying pathological amyloid deposition and corresponded to intermediate-to-high CERAD and Thal stages, with substantial agreement with visually determined scan positivity across diverse tracer modalities and separate neuropathological cohorts [9].

### 4.2. The Intermediate Gray Zone (10–30 CL)

**Figure 1:**
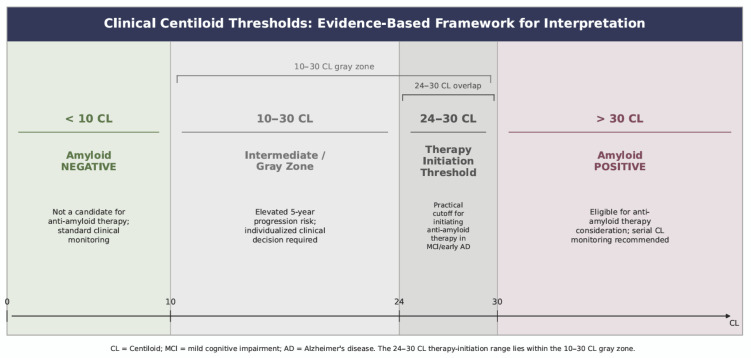
Clinical Centiloid Thresholds: Evidence-Based Framework for Interpretation.

While expert consensus and emerging cohort data recognise the clinical significance of this range, values from 10 to 30 CL constitute a clinically problematic intermediate, or “gray,” interval that has attracted considerable recent scrutiny. This interval resists simple binary categorization, as it reflects a continuum extending from sparse neuritic plaque deposition, corresponding to Thal phases 1 to 2, toward more moderate plaque burden, and includes both likely stable individuals and those at increased risk of progression to symptomatic AD. This interval has prognostic relevance irrespective of clinical status. In the AMYPAD Prognostic and Natural History Study (PNHS), individuals within this spectrum show a higher risk of cognitive decline than those with values below 10 CL, including participants who were cognitively intact at baseline [9]. The clinical interpretation of the established diagnostic, intermediate, and treatment-initiation Centiloid thresholds is summarized in Figure 1.

### 4.3. Concordance with CSF Biomarkers and Visual Read

An expanding corpus of research has assessed the agreement of CL quantification with CSF biomarker profiles and expert visual interpretation of PET imaging. Within this body of work, a 2025 study by Cerman and colleagues found 93% concordance of CSF A-beta42/40 ratios derived from automated platforms and amyloid PET at a threshold of 12 CL, consistent with the donanemab discontinuation criterion and with early histopathological detection [20].

The Centiloid threshold that corresponds to a visually positive amyloid PET scan is not consistent across all studies. Reported cutoffs range from about 17 CL when scans are interpreted by highly expert evaluators to about 40 CL in routine clinical practice, although the value most consistently associated with visual positivity is around 25 CL. The overall visual interpretation by local readers also demonstrates strong concordance with Centiloid status, with a kappa value of 0.85 and an overall agreement rate of 92.3%. This robust concordance was likewise evident across distinct disease stages [21].

## 5. The Centiloid Scale in Anti-Amyloid Therapy

### 5.1. Overview of FDA-Approved Anti-Amyloid Therapies

The approval of lecanemab and donanemab represented a major milestone in Alzheimer’s disease therapeutics, as they were the first disease-modifying drugs to demonstrate a statistically significant attenuation of clinical decline in large Phase III trials. Both drugs are humanized IgG1 monoclonal antibodies, but they target distinct forms of aggregated Aβ, with lecanemab primarily binding soluble Aβ protofibrils, whereas donanemab targets amyloid plaques containing N-terminal pyroglutamate-modified Aβ [7]. Both agents have also shown substantial amyloid clearance, which was measured using Centiloid units [7,22].

### 5.2. Treatment Eligibility and the 24–30 CL Threshold

Following regulatory approval of these agents, a major unresolved issue was the determination of an appropriate quantitative CL cutoff for treatment eligibility, particularly for cases that fell within the low-confidence equivocal range of visual interpretation. The FDA prescribing information for lecanemab and donanemab does not stipulate a defined quantitative centiloid cutoff for treatment eligibility and instead requires only documented amyloid positivity established by PET imaging or cerebrospinal fluid analysis [23,24].

To establish an optimal CL cutoff for treatment eligibility, the Alzheimer’s Association Research Roundtable, composed of leading global experts in dementia across neurology, nuclear medicine, and radiology, assembled in May 2024. Based on expert consensus, a practical consensus range of 24–30 CL was accepted as the cutoff for commencing anti-amyloid therapy in patients with MCI attributable to AD or mild AD dementia. It should be noted that this range reflects expert consensus rather than a prospectively validated cutoff, and its application in individual clinical decisions requires careful judgment [23].

The evidence-based support for this range is inferential yet concordant, as a CL value of 26 provided the highest overall predictive value for progression to dementia over 6 years in a longitudinal memory-clinic cohort. The concordance around CL = 26 +/− 7 has also been reported across independent lines of evidence and several endpoints, including subsequent dementia, PET change, CSF biomarkers, and histopathology [16].

Moreover, although CSF tau/Abeta42 ratios have identified optimal PET thresholds of approximately 28–30 CL, CSF Abeta42 alone has supported a lower threshold near 12 CL, consistent with early amyloid alteration rather than established amyloid pathology [25].

Institutional heterogeneity further impedes consensus, as visual interpretation, reader expertise, tracer selection, processing pipelines, reference-region selection, scanner harmonization, and site-specific implementation may all influence the relationship between visual positivity and quantitative values [26]. These reader- and site-related effects are supported by findings from the Imaging Dementia-Evidence for Amyloid Scanning (IDEAS) study, in which community-based visual reads showed 86% concordance with quantitative amyloid status, compared with approximately 93% to 95% agreement reported in expert-reader studies [23].

Interpretation of discordant findings is further complicated by the fact that these ranges have not yet been prospectively validated as universal treatment-initiation thresholds across tracers, scanners, software pipelines, demographic groups, and real-world clinical settings. Accordingly, prospective multicenter validation studies are required to determine whether patients within the consensus range derive therapeutic benefit comparable to that observed in patients with higher amyloid burden, and whether this range demonstrates consistent performance across institutional workflows and patient populations [9].

### 5.3. Lecanemab (CLARITY-AD Trial): Centiloid as an Efficacy Endpoint

Under the CLARITY-AD trial framework, Centiloid units were included as a prespecified secondary endpoint. In this phase III study, 1795 individuals with either MCI or mild AD dementia were randomly assigned to receive lecanemab at a dose of 10 mg/kg by intravenous infusion every 2 weeks or placebo over an 18-month period. Treatment with lecanemab produced a substantial reduction of 55 CL from baseline, compared with a slight increase of 3 to 4 CL in the placebo group, yielding a between-group difference of −59.12 CL (*p* < 0.0001) [7].

Notwithstanding, a pivotal safety consideration in current analyses remains the limited utility of baseline amyloid burden, as measured by Centiloid, in predicting Amyloid-Related Imaging Abnormalities-Edema (ARIA-E) risk. On the contrary, the APOE ε4 genotype, particularly homozygosity, and baseline indicators of cerebral amyloid angiopathy, such as microhemorrhages, continue to be the most consistently identified risk factors. Although preliminary subgroup analyses indicate no consistent dose–response association between initial amyloid burden and ARIA risk, this relationship remains incompletely defined and warrants further validation [7,27,28,29].

### 5.4. Donanemab (TRAILBLAZER-ALZ2 Trial): The ‘Treat-to-Clear’ CL Strategy

In the Phase III TRAILBLAZER-ALZ 2 trial, 1736 participants with early symptomatic AD randomly received either donanemab or placebo for 76 weeks. Eligible participants had confirmed amyloid pathology (CL ≥ 37) and positive tau PET scans. Donanemab was given intravenously once monthly at 700 mg, with escalation to 1400 mg. The trial adopted a notably intensive amyloid-reduction strategy, using serial amyloid PET scans to monitor response, with donanemab discontinued and replaced with placebo once amyloid clearance was verified [30].

Donanemab demonstrated striking, near-complete amyloid clearance, lowering the amyloid load by 87 CL from a baseline of 107 CL over 76 weeks in the low-to-medium tau subgroup, which represents the population with the most favorable prognosis. The treatment cessation thresholds were defined as a CL value below 11 on a single scan or below 25 on two consecutive scans. These thresholds were reached by approximately 40% of treated participants by 24 weeks and by 71% by 52 weeks, which highlights the vigorous amyloid-reducing efficacy of this agent [30].

Evidence regarding the persistence of amyloid clearance after treatment cessation remains limited. In post hoc analyses of TRAILBLAZER-ALZ, participants who achieved amyloid reduction to less than 11 CL by week 24 and subsequently discontinued donanemab sustained amyloid clearance over the subsequent year. The mean rate of amyloid reaccumulation was estimated at 0.02 CL per year. Exposure-response modeling further indicated that amyloid burden would require a median of 3.9 years to increase from 11 CL to the 24.1 CL amyloid-positivity threshold, corresponding to a projected reaccumulation rate of approximately 3.4 CL per year. These findings provide a biological rationale for a treat-to-clear approach, although they remain exploratory and hypothesis-generating [31].

## 6. Clinical Workflow for Centiloid Integration in Practice

### 6.1. Pre-Treatment Assessment

A thorough clinical evaluation establishes the foundation for anti-amyloid treatment initiation. Although amyloid PET with CL quantification serves as the principal imaging biomarker for treatment eligibility, it should be interpreted within a multimodal framework, as outlined in current appropriate use criteria and the 2024 expert consensus. This can be organized into the following five steps.

First, clinical staging is used to confirm MCI due to AD or mild AD dementia (CDR 0.5–1.0, MMSE 20–30), thereby excluding patients with severe dementia from current treatment protocols.

Second, the amyloid pathology is confirmed using either CSF analysis or amyloid PET with Centiloid quantification. Amyloid PET may be preferred in treatment-eligible patients because it permits direct visualization of plaque distribution and enables quantitative monitoring of amyloid clearance.

Third, Centiloid values are best contextualized within the established clinical framework (as discussed in Section 4.2 and Section 5.2), with the report noting whether the CL falls below the amyloid-negative threshold, within the indeterminate range, or clearly above 30 CL, consistent with high-confidence amyloid positivity. Fourth, as part of the pretreatment safety evaluation, a baseline brain MRI obtained within 12 months is recommended to exclude patients who may be at increased risk of treatment-related brain complications. These MRI red flags include more than four cerebral microbleeds, macro hemorrhage exceeding 1 cm, cortical superficial siderosis, existing ARIA, significant vascular disease, severe leukoaraiosis (Fazekas grade 3 or extensive Confluent White Matter Hyperintensities (C-WMH)), multiple lacunar infarcts, or large infarcts. In addition, APOE ε4 genotyping can be performed to guide counseling regarding ARIA risk.

Finally, for patients receiving anti-amyloid therapy, a standardized amyloid PET report is recommended that includes (a) the visual interpretation result (positive, negative, or indeterminate), (b) the CL value, (c) its interpretation with respect to established thresholds, and (d) a recommendation for clinical management.

### 6.2. On-Therapy Monitoring and the CL as a Treatment Biomarker

At present, longitudinal clinical assessment and serial brain MRI surveillance for the detection of ARIA remain the cornerstones of monitoring during anti-amyloid therapy. The application of amyloid PET and subsequent Centiloid scoring differs among trials and may remain discretionary in some. In lecanemab studies, follow-up PET is commonly conducted at 12–18 months to verify amyloid clearance. By contrast, donanemab’s “treat-to-clear” strategy requires serial PET approximately every 6–8 months to assess whether the amyloid clearance criteria defined in Section 5.4 have been met [32].

### 6.3. Critical Caveats for Reporting Radiologists

Several practical considerations merit attention from radiologists when preparing amyloid PET reports that incorporate CL values, as outlined below.

First, CL quantification serves as a complement rather than a substitute for visual interpretation, which is best conducted independently before consideration of the quantitative output. Mask placement errors, particularly misplacement of the cerebellar reference region, can lead to visual–quantitative discordance and must therefore be identified through careful review of the image-processing output.

Second, there is no single CL cutoff that can be applied to all clinical scenarios.

Third, an elevated CL value alone does not establish treatment eligibility, and irrespective of the CL level, APOE ε4 genotyping and MRI-based exclusion of significant cerebrovascular disease remain essential before initiation of anti-amyloid therapy.

Fourth, amyloid positivity is not a reliable index of dementia severity. As amyloid deposition occurs early and remains relatively stable after clinical symptom emergence, CL values show only limited association with cognitive status at a given time point. By contrast, tau and neurodegeneration biomarkers align more closely with the extent of contemporaneous cognitive impairment.

Finally, standardized reports for therapy monitoring are recommended to document the date of the comparison scan, the baseline CL, the follow-up CL, the absolute change in CL, and the clinical interpretation with respect to discontinuation criteria.

### 6.4. Author’s Suggestion for Reporting and Using Centiloid Score in Alzheimer’s Disease Diagnosis and Work-Up

Supplement S1 presents a concise template for integrating baseline and follow-up Centiloid scores into radiology reports for use by radiologists and nuclear medicine specialists.

### 6.5. Examples

**Figure 2:**
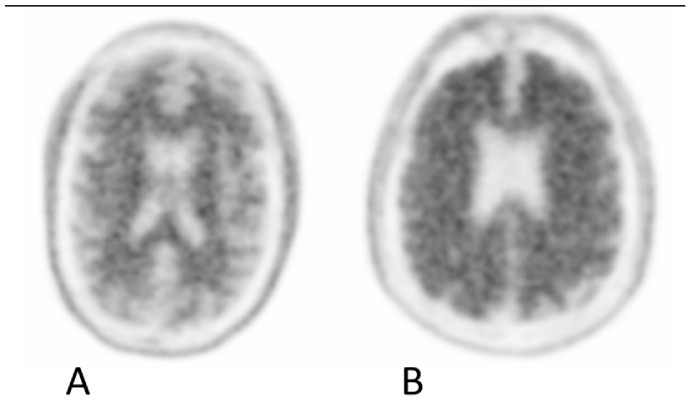
Visual interpretation of amyloid PET (Amyvid). (**A**) Negative scan. Preserved gray-white matter differentiation and predominant white matter uptake can be appreciated. (**B**) Positive scan, showing loss of gray-white matter contrast. This represents a binary visual assessment without quantification. Subsequent CL calculation was −10 for patient A, which indicates an amyloid burden lower than that observed in young healthy individuals, and 111 for panel B, which demonstrates an amyloid burden higher than that of patients with moderate AD symptoms. Of note, CL can be less than 0 or more than 100.

**Figure 3:**
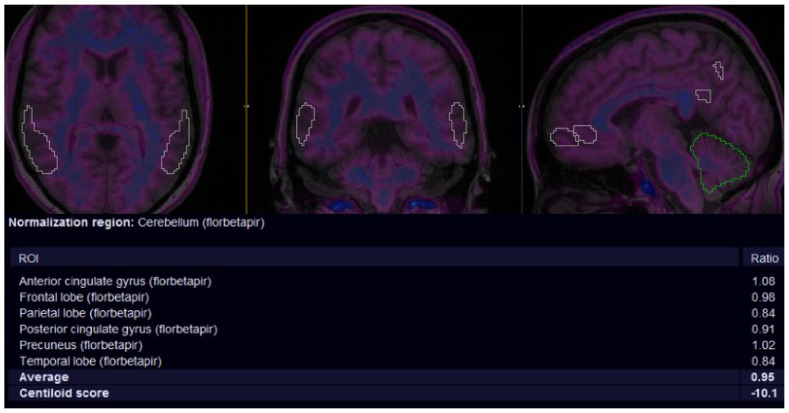
The CL for case A was calculated using automated software. The green region denotes the reference region (cerebellum). The white regions indicate the measurement areas, including the frontal, parietal, and temporal lobes, as well as the anterior and posterior cingulate and precuneus gyri. The final CL is derived from the aggregate values of the different regions of interest (ROIs). Although individual ROIs can be reported and tracked separately, their clinical relevance is not well established at present. As illustrated above, the ROIs are selected automatically by the software. Different software packages may select slightly different locations, which may result in minor variations in the CL value. Use of a single software package is recommended for longitudinal follow-up in each patient during treatment.

Representative examples of negative and positive amyloid PET scans based on visual gray-white matter contrast are shown in Figure 2. The automated Centiloid quantification output, including the cerebellar reference region and cortical regions of interest used to calculate the aggregate CL value, is shown in Figure 3.

### 6.6. Published Representative Longitudinal Examples of Serial Amyloid PET During Lecanemab Therapy

Real-world serial amyloid PET studies have begun to characterize longitudinal CL trajectories during lecanemab therapy. Iizuka et al. [33] reported a prospective observational cohort of 23 patients with early-stage Alzheimer’s disease treated with lecanemab 10 mg/kg every 2 weeks. These patients underwent serial 18F-flutemetamol amyloid PET at baseline, 6 months, and 12 months, with amyloid burden quantified in CL units. Their clinical status was assessed longitudinally using cognitive and functional measures. Two representative examples from that study are summarized in Table 2. They demonstrate that amyloid clearance trajectories may vary substantially between patients receiving the same treatment regimen, ranging from near-complete amyloid clearance to slower reduction over the same follow-up period [33].

Individual cognitive outcomes for the two representative examples were not reported separately; therefore, the findings are best understood as longitudinal PET examples rather than evidence of patient-level clinical response. At the cohort level, cognitive decline occurred in some patients despite substantial amyloid reduction. This reinforces that CL change warrants interpretation alongside cognition, function, MRI surveillance for amyloid-related imaging abnormalities, and complementary biomarkers when available [33].

## 7. Technical Limitations and Pitfalls

### 7.1. Methodological Sources of Variability

Several technical factors may contribute to variability in CL measurements and thereby distort clinical decision-making in borderline cases, as discussed below.

Errors in cerebellar reference region selection constitute the most consequential source of systematic bias. SUVR and CL values may spuriously increase because of white matter contamination in the cerebellar reference region. This effect may be more pronounced in patients with posterior fossa pathology. In the case of flutemetamol, the white matter reference region may also be affected by periventricular lesions [26,34,35].

The next factor is the effect of partial volume averaging of gray matter signals with surrounding cerebrospinal fluid in patients with significant brain atrophy. This can underestimate true tracer uptake, accompanied by falsely low CL values, which may result in misclassification of patients with significant pathology as negative or equivocal. This issue appears particularly relevant in later-stage patients undergoing treatment monitoring, in whom atrophy is more pronounced [36,37].

Motion artifacts caused by patient movements during PET acquisition are another prevalent challenge that can blur cortical boundaries, diminish gray-white contrast, and reduce the precision of SUVR estimation. Motion correction methods available in most contemporary PET systems warrant routine application [38].

The obtained CL values from different PET scanner platforms and imaging sites may exhibit systematic variation even in the same patient. This introduces the next challenge, namely inter-scanner variability, which may reflect differences in point spread function, reconstruction algorithms, and attenuation correction. Cross-scanner harmonization procedures are recommended for multi-site studies and are increasingly incorporated into commercial software packages [39].

The off-target white matter binding of flutemetamol represents an additional source of visual–quantitative discordance. This is more pronounced in cases with extensive periventricular white matter lesions, which necessitates careful correlation of CL quantification output with visual readout [24,40].

### 7.2. Biological Limitations

Beyond methodological considerations, CL measurement has inherent biological constraints.

The CL scale measures fibrillar amyloid plaque density but is not a direct reflection of soluble oligomeric Aβ species. These are increasingly recognized as the neurotoxic forms of amyloid most directly linked to synaptic dysfunction and cognitive impairment. Accordingly, because lecanemab targets soluble protofibrils, its therapeutic effects may be partly dissociable from the fibrillar plaque burden indexed by CL [41].

Moreover, as an inherent limitation of currently available PET tracers, amyloid PET and the CL scale cannot distinguish neuritic plaques, the lesion most pertinent to AD diagnosis, from diffuse plaques. The latter type of plaques also occurs in cognitively unimpaired aging and may exert distinct functional effects [41].

### 7.3. Centiloid in Prevention Trials

Emerging evidence suggests that CL thresholds may be applicable in cognitively unimpaired populations for prevention purposes, though prospective validation is still ongoing. The AHEAD 3-45 study represents the leading ongoing prevention trial to explicitly incorporate CL as an eligibility criterion in cognitively unimpaired individuals [42]. This lecanemab trial enrolls cognitively unimpaired adults into two strata based on CL: the A3 cohort, defined by intermediate amyloid levels of approximately 20–40 CL, and the A45 cohort, defined by elevated amyloid levels above 40 CL. Its design embeds CL directly into preventive medicine, with implications for population-level amyloid screening and early intervention. Pending prospective validation from ongoing prevention trials, CL reporting in cognitively unimpaired individuals may become relevant in selected research or clinical prevention settings.

### 7.4. The Relationship Between Amyloid Clearance and Clinical Benefit: An Ongoing Debate

In the pivotal CLARITY AD trial, lecanemab reduced 18-month Clinical Dementia Rating Sum of Boxes (CDR-SB) decline by approximately 27%, which corresponded to an absolute between-group difference of −0.45 points. Similarly, in TRAILBLAZER-ALZ 2, donanemab slowed CDR-SB progression by 36% in the low- and medium-tau population, with an absolute between-group difference of −0.67 points, and by approximately 29% in the combined population [7,30].

Although these effects were statistically significant, their clinical meaning at the individual-patient level remains debated. Reported minimal clinically important difference (MCID) estimates for the CDR-SB are approximately 1 point in MCI and 2 points in mild AD dementia, which are larger than the mean treatment-placebo differences reported in these pivotal trials. Therefore, group-level slowing of decline should not be assumed to indicate clinically meaningful benefit for every treated patient [43,44].

Responder analyses and within-patient change analyses have been proposed as more clinically interpretable alternatives to mean group differences. Care-partner-informed thresholds for meaningful within-patient change on the CDR-SB may also help contextualize individual-level treatment response [45,46].

Post hoc analyses of donanemab suggest that complete amyloid clearance may be associated with slower subsequent tau accumulation, particularly in later-affected cortical regions. However, amyloid clearance does not necessarily indicate absence of cognitive decline at the individual patient level [31].

Consistent with this distinction, plasma p-tau217 has been reported to associate strongly with AD-attributable memory deficits, and tau PET has outperformed amyloid PET as a predictor of future cognitive progression in head-to-head analyses [47,48].

Taken together, these findings support interpreting Centiloid reduction as evidence of amyloid target engagement and, in the regulatory context, as a surrogate endpoint, rather than as a guaranteed predictor of individual cognitive preservation [49,50].

### 7.5. Managing Discordant Biomarker Findings in Clinical Practice

Cross-platform comparisons show that biomarker agreement is high but incomplete. In validation studies, the ALZPath plasma p-tau217 assay agrees with amyloid PET in 95.8% of individuals with low p-tau217 values, but agreement fell to 86.3% among those with high p-tau217 values. CSF-PET studies show similar performance, with reported concordance rates of 93% for Lumipulse CSF Abeta42/40 and 92% for Lumipulse p-tau181/Abeta42. Thus, a minority of patients will have discordant plasma, CSF, and PET results, particularly near diagnostic thresholds [20,51].

Discordance is most relevant when CL values fall near clinical decision thresholds. A patient with low or gray-zone amyloid PET signal but clearly elevated plasma p-tau217 may be in an early stage of AD biology, before amyloid PET has reached a high-confidence positive range [23,52].

Conversely, borderline-positive CL values with normal tau biomarkers may indicate amyloid accumulation before detectable tau abnormality, particularly in older patients. However, if cognitive impairment is greater than expected for the degree of amyloid burden, or if biomarker results are internally inconsistent, mixed pathology or a non-AD cause should remain in the differential diagnosis [20,53].

Current multimodal frameworks, including the 2024 Alzheimer’s Association criteria and the 2025 amyloid and tau PET appropriate-use criteria, support amyloid PET, CSF Abeta42/40, and validated plasma assays such as p-tau217 as core biomarkers. Amyloid or tau PET is most useful when blood or CSF results are intermediate, discordant, or inconsistent with the clinical syndrome. In such cases, plasma p-tau217 should not be used as the sole basis for diagnostic classification or treatment eligibility [54,55,56].

In clinical practice, intermediate or discordant blood-biomarker results warrant cautious evaluation and confirmation with CSF biomarkers or amyloid PET when the result would affect diagnosis, counseling, or treatment eligibility. Repeat biomarker assessment may also be reasonable when the clinical syndrome and biomarker profile remain discordant [57].

### 7.6. Practical Feasibility of Serial Amyloid PET and the Role of Alternative Biomarkers

Applying the amyloid clearance criteria defined in TRAILBLAZER-ALZ 2 (Section 5.4), approximately 80% of participants in the low- and medium-tau population achieved amyloid levels below 24.1 CL by week 76. In TRAILBLAZER-ALZ 4, the median time to amyloid plaque clearance with donanemab was 359 days [30,58,59]. Serial amyloid PET is therefore biologically informative, but routine implementation is limited by cost, radiation exposure, tracer availability, scanner capacity, and reimbursement variability. Most economic studies have evaluated amyloid PET for diagnosis rather than repeated PET monitoring during anti-amyloid treatment [56,59,60].

Inter-pipeline variation in CL estimation further complicates clinical implementation. In a comparative analysis of 210 [18F] flutemetamol PET scans, three regulatory-approved commercial software packages and four research pipelines demonstrated strong group-level concordance for continuous CL measurements (R^2^ >= 0.93). However, concordance was substantially weaker at the individual-scan level. Between pipeline differences yielded 95% limits of agreement ranging from 12 to 23 CL. These discrepancies were most clinically consequential for scans near amyloid-positivity thresholds, where they could change binary classification [61].

In 2023, the U.S. Centers for Medicare and Medicaid Services removed the national coverage determination for beta-amyloid PET, ended coverage with evidence development, eliminated the previous one-scan limit, and left coverage decisions to Medicare Administrative Contractors [62]. This policy change may improve access to amyloid PET in some regions. Conversely, it may also increase geographic and payer-related variation in coverage, particularly when PET is used repeatedly for treatment monitoring rather than for diagnostic assessment. In a separate treatment-burden model of disease-modifying Alzheimer therapies, a fixed-duration infusion strategy required two PET scans to confirm amyloid clearance, and monitoring for initial and sustained amyloid clearance accounted for 12,178 USD in additional medical costs [63]. These data suggest that CL-guided treat-to-clear strategies may reduce drug-exposure duration but can also shift part of the economic burden to imaging, monitoring, and follow-up infrastructure.

Plasma biomarkers may eventually reduce the need for repeated PET scans. In a Treatment-related amyloid clearance (TRAC) framework analysis, declining amyloid PET signal during treatment correlated with increasing plasma Abeta42/40 and decreasing plasma p-tau species. Nevertheless, no plasma-based stopping threshold has yet been prospectively validated for clinical use [58,64,65].

Plasma phosphorylated tau 217 and amyloid-beta 42/40-based blood biomarkers show good diagnostic concordance with amyloid PET, with reported stand-alone sensitivity and specificity of approximately 84% to 90%. Despite this, two-cutoff approaches still leave an intermediate group requiring confirmatory amyloid PET or CSF testing in approximately 10% to 40% of individuals. Performance is also less robust in early or preclinical populations, where lower Alzheimer’s disease prevalence reduces positive predictive value and increases the proportion of indeterminate results. These biomarkers also do not yet reliably quantify continuous amyloid PET burden or monitor plaque clearance after immunotherapy. In TRAILBLAZER-ALZ, change in Centiloid correlated only moderately with change in plasma p-tau217 (R = 0.484), and p-tau217 responses varied substantially even among participants with approximately 100 Centiloid PET reduction. Given the modest amyloid reaccumulation rates observed after treatment cessation (Section 5.4), whether blood biomarkers can guide retreatment timing remains uncertain [66].

Until such thresholds are validated in adequately powered therapeutic trials, amyloid PET remains the reference standard for documenting amyloid clearance during anti-amyloid therapy [6,67,68].

## 8. Conclusions

The Centiloid scale has evolved from a research standardization instrument into a clinically essential biomarker in the management of Alzheimer’s disease. Its ability to generate tracer-independent, universally interpretable measures of amyloid burden has supported three major advances. These include more precise classification of amyloid status beyond binary visual interpretation, evidence-based determination of treatment eligibility, and objective assessment of amyloid clearance during and after anti-amyloid therapy.

The FDA approval of lecanemab and donanemab has further redefined amyloid PET from a diagnostic modality into a therapeutic biomarker. This shift is exemplified by the substantial amyloid reductions demonstrated in the CLARITY-AD and TRAILBLAZER-ALZ 2 trials, with the latter pioneering a “treat-to-clear” strategy with explicit CL-based stopping criteria. These developments underscore the need for neuroradiologists and nuclear medicine physicians to develop and maintain proficiency with CL quantification as part of their clinical reporting.

Future work aimed at enhancing the precision of AD staging and therapeutic monitoring could prioritize the integration of CL with plasma biomarkers, AI-driven quantification methods, and tau PET. As prevention trials start to recruit cognitively unimpaired individuals, the amyloid PET may become increasingly routine practice, not only in diagnosis but also as an ongoing, dynamic marker of disease course and treatment response.

## Figures and Tables

**Table 1:** FDA-Approved Amyloid PET Tracers and Their Key Characteristics.

Tracer (Brand Name)	SUVR Reference Region	Sensitivity/Specificity	CL Conversion
18F-Florbetapir (Amyvid)	Whole cerebellum	~92–96%/~90–100%	Linear transformation vs. PiB
18F-Florbetaben (NeuraCeq)	Whole cerebellum	~98%/~89%	Linear transformation vs. PiB
18F-Flutemetamol (Vizamyl)	White matter/cerebellum	~93%/~93%	Linear transformation vs. PiB
11C-PiB (Pittsburgh Compound B)	Cerebellum (anchor tracer)	Reference standard	0 CL = young controls; 100 CL = mild-mod AD

*Abbreviations: SUVR, standardized uptake value ratio; CL, Centiloid; PiB, Pittsburgh Compound B; AD, Alzheimer’s disease. All fluorinated tracers are approved by the FDA for clinical use. PiB is available only for research.*

**Table 2:** Published representative longitudinal amyloid PET examples from Iizuka et al. (2026) [33].

Published Example	Treatment	Baseline CL	6-Month CL	12-Month CL	Imaging Interpretation
Woman in her 80 s	Lecanemab	112.7	17.6	−9.9	Rapid near-complete amyloid clearance
Woman in her 50 s	Lecanemab	91.6	77.2	63.8	Slower amyloid clearance despite the same treatment protocol

## Data Availability

No new data were created or analyzed in this study. Data sharing is not applicable to this article.

## Supplementary Materials

The following supporting information can be downloaded at https://www.mdpi.com/article/10.3390/diagnostics16131989/s1, Supplement S1: Template for radiology report.
