# Supplementary material for: The Centiloid Scale in Amyloid PET Imaging: Current Role in Alzheimer’s Disease Diagnosis, Treatment Planning, and Monitoring During Anti-Amyloid Therapy: A Clinical Perspective
Source: Diagnostics (Basel). 2026 Jun 26;16(13):1989. doi: 10.3390/diagnostics16131989 (PMC13359828; doi:10.3390/diagnostics16131989)
Supplement: Supplementary file 1 [file diagnostics-16-01989-s001.zip › diagnostics-4294080-supplementary.pdf]

Supplement S1.

Template for radiology report

[1] Amyloid PET Brain: _________ (POSITIVE / NEGATIVE) for elevated cortical amyloid burden.

[2] Centiloid value:

Initial PET_______ CL (SUVR: _______ ; Tracer: _________ ; Software: ___________).

[3] CL interpretation: ________________________________ (Negative / Intermediate / Low Positive / Positive).

[4] Visual–quantitative concordance: _________________________ (Concordant / Discordant).

[5] Recommended follow-up amyloid PET: __________________________ (12 months for donanemab / 18 months for lecanemab; per clinical protocol).

For the follow-up PET, this should be added:

[6] Follow up PET ______ CL (SUVR: _______ ; Tracer: _________ ; Software: ___________).

[7] Interval change in CL ------

*** Please note that the role of different software is not clearly known. For comparison, using the same software is recommended.

Changes in Centiloid score (to be used on follow-up scan):
